# Influence of the type of facial pattern on the outcome of treatment of patients with obstructive sleep-disordered breathing with an intraoral appliance

**DOI:** 10.1016/j.bjorl.2026.101890

**Published:** 2026-10-05

**Authors:** Henrique Cabrini Moreira, Rafael de Andrade Balsalobre, Maximiana Cristina de Souza Maliska, Milena de Almeida Torres Campanholo, Renato Prescinotto, Danilo Anunciatto Sguillar, Fernanda Louise Martinho Haddad

**Affiliations:** Universidade Federal de São Paulo, Department of Sleep Apnea and Otolaryngology, São Paulo, SP, Brazil

**Keywords:** Sleep apnea, obstructive type, Advance device, Mandibular, Retrognathia, Snoring

## Abstract

**Objectives:**

To evaluate whether the type of facial pattern obtained from standardized photographs influences the success of treatment with Oral Appliance (OA) in a group of patients with Obstructive Sleep Disorders (OSD), and to verify the agreement between three examiners regarding the classification of the type of facial pattern.

**Methods:**

Fifty patients with Obstructive Sleep Apnea (OSA) underwent systematic evaluation of the face using standardized photographs to characterize the type of facial pattern. The patients were examined, blindly and consecutively, by three dental surgeons.

**Results:**

There was strong agreement between the examiners about the type of facial pattern (κ = 0.899). OA success rates were similar between patients with facial pattern I and II.

**Conclusion:**

The type of facial pattern, I and II, did not influence the success of OA. Both facial patterns can benefit from this, and the test was reproducible with strong inter-rater agreement.

**Levels of evidence:**

Level 2.

## Highlights

• The intraoral mandibular advancement Oral Appliance (OA).
• Standardized photographs influence the success of treatment with OA.
• Facial patterns can benefit from a test that is reproducible between evaluators.
• Easily performed by photographic evaluation or facial inspection, with low cost.
• The analysis can be performed in a medical or dental office.

## Introduction

Obstructive Sleep Apnea (OSA) is characterized by the repetitive collapse of the upper airways during sleep with an intermittent hypoxia that causes sleep fragmentation in an attempt to recover the patency of the normal airflow during breathing.^1^ It was first described in 1993 by the American Academy of Sleep Medicine (AASM), and since then it has been updated regarding its criteria and treatments.^1^^,^^2^

The risk factors associated with OSA involve age, male gender, increased Body Mass Index (BMI), increased neck Circumference (CC) measurement, and Upper Airway (UAV) and craniofacial changes.^3^^,^^4^ Abnormal anatomical characteristics that can be present in individuals with OSA are: thick soft palate, median tonsillar pillars, obstructive hypertrophy of the palatine tonsil, long and/or thick uvula, and large tongue.^5^ Added to this are craniofacial deformities such as inferior position of the hyoid bone, greater flexion of the skull base, maxillary hypoplasia and mandibular retrognathia.^6^

During the extra and intraoral physical examination, it is important to evaluate the presence of maxillary atresia, Angle's Class II dental occlusion and disproportions between the maxilla and mandible, and changes in the distances obtained between the hyoid bone, thyroid, cricoid and chin are the parameters most correlated to the disease.^3^^,^^7^^,^^8^

According to the AASM, Oral Appliance (OA) is preferably indicated in patients with mild and moderate OSA and may be indicated in severe cases when treatment with Continuous Positive Airway Pressure (CPAP) fails or is not well tolerated by the patients.^9^ Its mechanism of action aims at reducing the VAS collapse by performing the mandibular protrusion and allowing the repositioning of the soft palate and tongue, which allows an increase in the velopharyngeal airway space and thus an airflow free of obstructions.^10^, ^11^, ^12^

It is known that in addition to clinical and polysomnographic characteristics, some craniofacial parameters obtained by imaging exams may influence the success of therapy.^13^ However, these methods require a specialized laboratory, expose the patient to radiation, and have a high cost, often making it unfeasible to systematically perform them in all patients with OSA.^6^^,^^14^ It is believed that findings related to mandibular hypoplasia are good predictors of treatment success with OA.

Currently, there is a trend to value facial characterization in facial pattern types, which can be easily performed by photographic evaluation or facial inspection, with low cost, can be performed in a medical or dental office, and without the need for radiation. Despite the advantages of the method, it is a subjective evaluation of the face that could suffer from inter-examiner influence or disagreement. In this context, it is believed that patients with facial pattern type 2, suggestive of mandibular retroposition, would respond best to therapy, but we know that many patients with OSA who would be potential candidates for treatment with OA may not present skeletal changes, and usually have facial pattern type.^1^^,^^9^

Thus, this study sought to evaluate the influence of facial type on OA treatment in patients with Obstructive Sleep-Disordered Breathing (OBS), as well as to verify the level of inter-rater agreement regarding the classification of facial pattern type.

## Methods

A retrospective study was carried out in a private sleep dentistry clinic during the year 2021 and the project was approved by the Research Ethics Committee of Department of Otolaryngology and Head and Neck Surgery at UNIFESP. We selected the medical records of patients seen from 2012 to 2021 who were diagnosed with OSD, according to the International Classification of Sleep Disorders (ICDS-3 TR)^2^ and were treated with OA.

The following criteria were employed for inclusion in the study: 1) Patients older than 18-years; 2) Both genders; 3) With baseline full night polysomnography within 6-months of the start of treatment with OA; 4) With polysomnography in use of the intraoral device full night after therapeutic advance or comfortable maximum with at least 6-months of titration and 5) With standardized photos of front and profile that allowed the characterization of the type of facial pattern (these photos were standardized in natural head position, NHP, which is a reproducible and physiological position). Exclusion criteria were: 1) Medical records with incomplete data, 2) No initial or final polysomnography using the OA, 3) Incomplete orthodontic documentation (without standardized cephalometric analyses like the rest of the patients), 4) Patients who consumed alcohol for more than 4-days a week, 5) Patients taking sedatives or muscle relaxants, and 6) Patients with decompensated clinical or psychiatric illnesses.

The evaluation protocol applied followed the analysis of demographic data, polysomnographic data, and subjective face evaluation. The demographic data used were: 1) Age; 2) Gender; 2) BMI. The polysomnographic data considered were: 1) Apnea and hypopnea index; 2) Percentage of oxygen saturation below 90%; 3) Minimum oxygen saturation; 4) Rate of awakenings per hour of sleep; and 6) Percentage of sleep phases N1, N2, N3, REM, both at baseline and with OA.

The subjective evaluation of the face was performed by three different evaluators with experience in the area (dental surgeons). The examinations were conducted prospectively and blindly, and there was no communication during the evaluation. In case of disagreement by a single examiner, the predominant facial pattern type was considered. In case of disagreement between the 3 examiners, a new analysis was performed to reach an agreement on the facial pattern to be chosen.

After data collection, the following assessments were made: 1) Description of the types of facial patterns; 2) Comparison of the pre and post-OA polysomnographic findings; 3) Comparison of the pre and post-OA polysomnographic findings between the different facial patterns; 4) Evaluation of the overall success rate and the comparison of the different success rates in each facial pattern, following different criteria (reduction of the AHI by 50% below 15 h, reduction of the AHI by 50% below 10 h and reduction of the AHI to values below 5 h.

### Description of facial pattern types

The evaluation was performed using standardized photos with the patient sitting in the Natural Head Position (NHP), a natural and reproducible position. Patients were photographed sitting with their gaze fixed to the horizon on the wall at eye level so that there is no unwanted tilting. The head was deprogrammed by asking them to look up and down and then to look at the point on the horizon. The following were analyzed: tegument facial profile, chin-neckline, mentholabial sulcus, zygomatic projection, and the height of the lower third. On the photos the distance between the true vertical line passing through the subnasal point was measured. This line is a reference to aid in the clinical diagnosis of skeletal alterations in facial development. The description of the characteristics adopted for facial patterns I, II and III were based on Capelozza’s classification and are shown in Table 1.^7^

**Table 1:** Characteristics for analysis of facial patterns.

Aspects analyzed	Pattern I	Pattern II	Pattern III
Malocclusion	None or dental only	Positive sagittal relationship between maxilla and mandible	Negative sagittal relationship between the maxilla and the mandible
Balance of the facial thirds	Balanced (mesofacial)	Dolichofacial, mesofacial or brachyfacial	Dolichofacial, mesofacial or brachyfacial
Facial profile	Straight or moderate degree of convexity	Convex	Concave
Zygomatic projection	Good and present	Normal or excessive creating infraorbital depression and marked nasolabial folds	Normal or deficient
Nasolabial angle	Straight, obtuse or acute	Acute	Obtuse
Chin-neck line	Normal, parallel to Camper's plane	Short with mandibular deficiency	Normal or increased
Chin-neck angle	Good	Open	Closed
Lower facial third height	In balance with the other thirds	Typically reduced	Typically increased

We still have the short or long face patients. Long-faced patients have excessive vertical facial growth, i.e., increased Anterior Inferior Facial Height (LAFH), which prevents passive lip seal. Excess exposure of anterior teeth at rest and of gingiva on smiling is notorious. Increased facial convexity. Deficient maxillary expression. Short chin-neckline with deficient sagittal expression of the maxilla and mandible, which show a predominantly vertical growth direction. Long and narrow face.

The short face patient, on the other hand, demonstrates a decrease in the lower third of the face that takes the compressive lip seal with accentuation of the periorbital ridges. It is frequent to observe lack of exposure of the anterior teeth at rest and little exposure in the smile. Deficiencies in vertical dimensions with compressed lips with little exposure of the upper incisors at rest and on smiling, their facial convexity is reduced. Adequate maxillary expression with a diminished lower third.

### Polysomnographic evaluation

Patients underwent full night polysomnography, performed in the laboratory (type 1) or at home (type 2), with monitoring of the electroencephalogram, electromyogram (mentonian region and anterior tibial muscles), electrocardiogram, airflow (nasal cannula associated with a pressure transducer and oronasal thermistor), respiratory effort (thorax and abdomen), Peripheral Oxyhemoglobin Saturation (SpO_2_), snoring, and body position.

Sleep staging was done according to the criteria proposed by Rechtschaffen and Kales (1968)^15^^,^^24^ and arousals, following the American Sleep Disorders Association ‒ ASDA criteria.^15^ The analysis of respiratory events was performed according to the criteria proposed by the AASM.^16^

### Agreement analysis

In order to describe the degree of agreement between the three examiners on facial pattern aspects by photography, as well as the agreement between the two diagnostic methods photography and cephalometry, we used the *kappa* index, which is based on the number of concordant answers between the examiners.

The inter-examiner agreement was assessed using the kappa concordance test. According to the Landis and Koch classification, a kappa index between 0.21 and 0.40 is considered “fair”, between 0.41 and 0.60 “moderate”, between 0.61 and 0.80 “substantial”, and finally, between 0.81 and 1.00 “almost perfect”.^17^

### Statistical analysis

The data analysis included descriptive statistics, mean, median, standard deviation, minimum value and maximum value for numeric variables, and number and proportion for categorical variables. To analyze the behavior of continuous variables, we considered the descriptive statistics, histogram and boxplot plots, and the specific test for the Shapiro Wilk^18^ theoretical assumption of normality. The comparison between two groups was done by Student's *t*-test or Mann-Whitney's test for numerical variables and Pearson's Chi-Square test or Fisher's exact test for categorical variables. The comparison between groups in the pre- and post-treatment periods with OA was performed by the Wilcoxon Test for numerical variables without normal distribution. The statistical analysis was performed using the IBM-SPSS Statistics software version 28 (IBM Corporation, NY, USA). Values of *p* < 0.05 were considered significant.

## Results

The initial sample was composed of 150 patient records that after applying the inclusion and exclusion criteria, only 50 patients met the criteria. Regarding demographic data, the sample was composed of a majority of males (*n* = 32–64 %), with a mean age of 49.61 ± 10.47 years and a BMI of 26 Kg/m^2^. As for facial pattern, half (*n* = 25–50 %) were standard type II, were standard type I (46%) and 02 were standard type III (4%). There were no patients characterized as short face and long face.

The comparative analysis of the entire sample as to the polysomnographic parameters before and after the use of OA are shown in Table 2, Table 3; it is observed that there was a statistically significant difference for the following findings 1) Reduction in Apnea-Hypopnea Index (AHI) (*p* < 0.001); 2) Increase in minimum oxyhemoglobin saturation (*p* < 0.001); 3) Reduction in the time of saturation (*p* = 0.025); 4) Reduction in the number of awakenings (*p* = 0.013) Reduction in the percentage of N1 (*p* = 0.011).

**Table 2:** Descriptive statistics of polysomnographic findings in patients before OA treatment.

	*N*	Mean	SD	Minimum	Perc.25	Perc.50 (median)	Perc.75	Maximum
AHI (n^o^/h)	50	21.42	13.78	1.00	11.53	17.20	28.53	66.80
O_2_ min (%)	50	83.32	5.62	65.00	80.75	84.00	87.25	92.00
T90	42	6.71	11.85	0.00	0.28	2.20	11.00	66.30
N1 (%)	50	12.14	12.39	0.60	3.95	8.85	15.70	70.40
N2 (%)	50	49.67	10.97	0.00	43.10	49.50	57.25	72.40
N3 (%)	50	16.26	8.88	0.00	9.00	16.00	23.68	31.90
REM (%)	50	19.99	6.94	0.00	15.28	19.65	26.60	34.90
Arousal (n^o^/h)	46	21.30	17.06	0.00	12.18	17.50	26.03	87.60

SD, Standard Deviation; Perc, Percentile; AHI, Apnea-Hypopnea Index; O_2_ min, Minimum Oxygen Saturation; T90, Percentage of sleep time below 90% oxygen saturation; N1, Stage 1; N2, Stage 2; N3, Stage 3; REM, Rapid Eye Movement stage.

**Table 3:** Descriptive statistics of polysomnographic findings in patients after OA treatment.

POST	*N*	Mean	SD	Minimum	Perc.25	Perc.50 (median)	Perc.75	Maximum
AHI (n^o^/h)	50	8.79	14.10	0.00	2.08	5.35	11.33	96.00
O_2_ min (%)	50	87.32	3.95	78.00	85.00	88.50	90.25	94.00
T90	40	3.84	8.92	0.00	0.00	0.30	2.40	44.00
N1 (%)	50	7.97	6.41	1.40	3.80	5.75	10.10	29.70
N2 (%)	50	52.35	10.09	26.60	46.45	52.35	56.85	77.30
N3 (%)	50	18.62	9.36	0.00	13.13	18.70	25.73	42.80
REM (%)	50	20.92	7.23	0.70	18.18	20.50	26.20	33.10
Arousal (n^o^/h)	46	15.12	14.87	0.00	7.53	11.25	18.00	87.60

SD, Standard Deviation; Perc, Percentile; AHI, Apnea-Hypopnea Index; O_2_ min, Minimum Oxygen Saturation; T90, Percentage of sleep time below 90% oxygen saturation; N1, Stage 1; N2, Stage 2; N3, Stage 3; REM, Rapid Eye Movement stage.

There were no short face and long face patients in the sample and only two patients with Pattern III, so the comparative analysis was performed only for patterns I and II. The comparative analysis of the polysomnographic parameters before and after the use of OA for patterns I and II are shown in Table 4, Table 5, and the findings of respiratory parameters (AHI and oxyhemoglobin saturation) were similar in both groups.

**Table 4:** Comparative analysis of polysomnographic findings between pre- and post-OA treatment periods for patients with facial pattern I evaluated from photographs.

Polysomnographic findings	Nº	Pattern		p-value
		Pre	Post	
AHI (n^o^/h)	23	14.3 (1.0–66.8)	5.0 (0.0–96.0)	0.001
O_2_ min	23	84.0 (70.0–92.0)	89.0 (78.0–93.0)	0.004
Sat. below 90%	18	1.1 (0.0–34.6)	0.2 (0.0–29.7)	0.224
Arousal (n^o^/h)	22	15.1 (0.0–87.6)	11.7 (0.6–87.6)	0.513
N1 (%)	23	8.4 (0.6–40.7)	5.3 (1.4–29.1)	0.224
N2 (%)	23	51.0 (33.5–72.4)	52.3 (26.6–77.3)	0.218
N3 (%)	23	16.6 (0.0–31.9)	17.8 (0.0–42.8)	0.341
REM	23	19.0 (0.0–34.9)	20.4 (0.7–33.1)	0.271

Wilcoxon test. Continuous variables are described as median (minimum‒maximum). AHI, Apnea-Hypopnea Index; O_2_ min, Minimum Oxygen Saturation, T90, Percentage of sleep time below 90% oxygen saturation; N1, Stage 1; N2, Stage 2; N3, Stage 3; REM, Rapid Eye Movement stage.

**Table 5:** Comparative analysis of polysomnographic findings between pre- and post-OA treatment periods for patients with facial pattern II evaluated from photographs.

Polysomnographic findings	Nº	Pattern II		p-value
		Pre	Post	
AHI (n^o^/h)	25	20.0 (2.8–53.1)	6.1 (0.0–26.9)	<0.001
O_2_ min	25	84.0 (65.0–91.0)	87.0 (78.0–94.0)	0.011
T90	17	2.3 (0.0–66.3)	0.6 (0.0–44.0)	0.094
Arousal (n^o^/h)	22	19.15 (2.2–75.6)	11.3 (0.0–47.3)	0.004
N1 (%)	25	9.4 (2.4–70.4)	7.5 (1.8–29.7)	0.017
N2 (%)	25	49.3 (0.0–66.8)	52.1 (30.2–66.2)	0.339
N3 (%)	25	15.4 (0.0–30.6)	20.3 (5.3–31.3)	0.074
REM	25	19.7 (9.0–30.0)	20.7 (8.1–33.1)	0.319

Wilcoxon test. Continuous variables are described as median (minimum-maximum). AHI, Apnea-Hypopnea Index; O_2_ min, Minimum Oxygen Saturation, T90, Percentage of sleep time below 90% oxygen saturation; N1, Stage 1; N2, Stage 2; N3, Stage 3; REM, Rapid Eye Movement stage.

The OA was analyzed by three different success criteria, these being 1) AHI ≥ 50% and AHI < 5/h with a success rate of 60%; 2) AHI ≥ 50% and AHI < 10 h, with a success rate of 56% and 3) AHI <5 h, with a success rate of 44% (Table 6, Table 7, Table 8). There were no statistically significant differences when comparing the different success criteria between type 1 and type 2 facial patterns.

**Table 6:** Comparative analysis between demographic data, baseline polysomnography, facial pattern and OA treatment success – success criterion 1 (AHI reduction ≥50% and AHI < 15/h).

		OA treatment success (AHI reduction ≥ 50% and AHI < 15/h)		
		Failure (*n* = 20)	Success (*n* = 30)	p-value
Age years^a^		48.50 ± 11.87	50.38 ± 9.53	0.543^c^
BMI, kg/m^2^^b^		26.49 (20.19–39.50)	27.30 (23.80–33.30)	0.659^d^
Gender, *n* (%)				
	Female	6 (30%)	12 (40%)	0.470
	Male	14 (70%)	18 (60%)	
AHI (basal)		12.10 (1.00–66.80)	22.05 (2.80–48.90)	**0.017**^d^
O_2_ min (basal)		84.00 (71.00–92.00)	84.00 (65.00–91.00)	0.377^d^
Sat. below 90% (basal)		2.10 (0.00–34.60)	2.80 (0.00–66.30)	0.879^d^
Facial pattern, *n* (%)				
	Facial pattern I	11 (55%)	12 (42.9%)	0.406
	Facial pattern II	9 (45%)	16 (57.1%)	

Continuous variables are described as mean ± standard deviation or median (range); Categorical variables are described in number (percentage).

Pearson's Chi-Square test.

BMI, Body Mass Index; AHI, Apnea Hypopnea Index.

a Available in 49 cases.

b Available in 26 cases.

c Student's *t*-test.

d Mann-Whitney test.

**Table 7:** Comparative analysis between demographic data, baseline polysomnography, facial pattern and success with OA treatment – success criterion 2 (AHI reduction ≥50% and AHI < 10/h).

		OA treatment success (AHI reduction ≥ 50% and AHI < 10/h)		
		Failure (*n* = 22)	Success (*n* = 28)	p-value
Age years^a^		47.91 ± 11.65	51.00 ± 9.40	0.309^c^
BMI, kg/m^2^^b^		26.87 (20.19–39.50)	27.00 (23.80–33.30)	0.893^d^
Gender, *n* (%)				
	Female	6 (27.3%)	12 (42.9%)	0.254^e^
	Male	16 (72.7%)	16 (57.1%)	
AHI (basal)		12.45 (1.00–66.80)	20.15 (2.80–48.90)	0.085^d^
O_2_ min (basal)		84.50 (71.00–92.00)	84.00 (65.00–89.00)	0.158^d^
T90 (basal)		2.10 (0.00–34.60)	2.80 (0.00–66.30)	0.715^d^
Facial pattern, *n* (%)				
	Facial pattern I	12 (54.5%)	11 (42.3%)	0.398^e^
	Facial pattern II	10 (45.5%)	15 (57.7%)	

Continuous variables are described as mean ± standard deviation or median (range); Categorical variables are described as number (percent).

BMI, Body Mass Index; AHI, Apnea-Hypopnea Index; O_2_ min, Minimum Oxygen Saturation, T90, Percentage of sleep time below 90% oxygen saturation.

a Available in 49 cases.

b Available in 26 cases.

c Student's *t*-test.

d Mann-Whitney test.

e Pearson's Chi-Square test.

**Table 8:** Comparative analysis between demographic data, baseline polysomnography, facial pattern and success with OA treatment – success criterion 3 (AHI < 5 h).

		OA treatment success (AHI < 5/h)		
		Failure (*n* = 28)	Success (*n* = 22)	p-value
Age years^a^		49.82 ± 10.64	49.33 ± 10.37	0.874^c^
BMI, kg/m^2^^b^		26.93 (20.19–39.50)	25.91 (21.67–33.30)	0.670^d^
Gender, *n* (%)				
	Female	9 (32.1%)	9 (40.9%)	0.522^e^
	Male	19 (67.9%)	13 (59.1%)	
AHI (basal)		17.60 (5.20–66.80)	17.20 (1.00–44.10)	0.482^d^
O_2_ min (basal)		83.00 (65.00–91.00)	84.50 (74.00–92.00)	0.367^d^
T90 (basal)		3.00 (0.00–34.60)	1.20 (0.00–66.30)	0.255^d^
Facial pattern, *n* (%)				
	Facial pattern I	12 (44.4%)	11 (52.4%)	0.585^e^
	Facial pattern II	15 (55.6%)	10 (47.6%)	

Continuous variables are described as mean ± standard deviation or median (range); Categorical variables are described as number (percent).

BMI, Body Mass Index; AHI, Apnea Hypopnea Index; O_2_ min, Minimum Oxygen Saturation, T90, Percentage of sleep time below 90% oxygen saturation.

a Available in 49-cases.

b Available in 26-cases.

c Student's *t*-test.

d Mann-Whitney test.

e Pearson's Chi-Square test.

There was strong agreement between the three examiners with respect to the facial pattern obtained by photography (*p* < 0.001). It is noteworthy that there were no patients characterized as short face and long face pattern in this sample. Table 9 shows the agreement values for the individual categories of the facial pattern. Table 10 shows the overall inter-rater agreement values for the facial pattern obtained per photograph.

**Table 9:** Agreement between examiners for individual categories of facial pattern evaluated from photographs according to Kappa agreement.

Facial Pattern Group	Examiner			Kappa	95% CI	p-value
	1	2	3			
Pattern I	23	23	22	0.919	0.759; 1.079	**<0.001**
Pattern II	25	25	27	0.893	0.733; 1.053	**<0.001**
Pattern III	two	two	1	0.793	0.633; 0.953	**<0.001**
Total	50	50	50			

CI, Confidence Interval.

**Table 10:** Overall agreement between examiners in relation to the facial pattern evaluated from photographs according to Kappa agreement.

	Examiner			Kappa	95% CI	p-value
Facial pattern	1	2	3			
Pattern I	23	23	22	0.899	0.753; 1.046	**<0.001**
Pattern II	25	25	27			
Pattern III	2	2	1			
Total	50	50	50			

CI, Confidence interval.

## Discussion

OSA has a prevalence for middle-aged males and females in the adult population worldwide. Tufik et al.^19^ conducted an epidemiologic study in the city of São Paulo with a prevalence of OSA of 32.9% in the general population, 40.6% in men and 26.1% in women.

Considering the facial patterns obtained, we found a higher prevalence of pattern type II, followed by type I. Among the 50 patients studied, 50% had a type II and 46% a type I facial pattern. This is justified by the prevalence of type I and II facial patterns being higher in the general population. Regarding populations with OSD, we found the type II pattern as a risk factor and the current literature emphasizes a higher rate of OSD in patients with this pattern with increased axial inclination of the incisors, 1 division of Angle's class II.^20^

Subjective facial evaluation has been gaining space due to its practicality and good reproducibility, since imaging exams require a specialized location, are expensive, and subject the patient to radiation, which is often not feasible for everyone.^21^ As in the study by Capelozza Filho et al.,^7^ we found a strong reproducibility in the characterization of facial patterns type I and II, which makes the method viable and reproducible, and can be used as an auxiliary method in the evaluation of these patients, not only by the dental specialist, but by other health professionals who work in the management of these patients, provided they are properly calibrated.

The evaluation between 3 examiners showed a satisfactory result regarding the standardization of facial identification by subjective physical examination, κ = 0.753 (substantial level). In the literature, we found agreement of *p* = 0.5, in the work of Reis et al.^22^ Therefore, predictability in interobserver agreement in characterizing the facial pattern becomes a feasible method in clinical practice.

Other studies in the literature attempt to relate the facial pattern with the OSA patient, but none of them portrays as this research, evaluating a subgroup of patients with indication for treatment with OA. According to the work of Scher et al. (2013),^25^ three criteria parameters were used, reduction of AHI ≥ 50% and AHI < 15 h, where 60% of patients showed success in OA therapy; reduction of AHI ≥ 50% and AHI < 10 h, with a 56% success rate and the stricter criterion of AHI < 5 h with 44% success rate.

It is evident that, even using the first criterion, the patient had a substantial decrease in AHI compared to the initial polysomnography. There was, then, a significant reduction between patients before and after the use of the OA when comparing the respiratory parameters of the initial and final polysomnographies, especially in the reduction of AHI, increased oxyhemoglobin (and reduced arousals, in both facial patterns.^23^

Two differential findings of this study are worth noting. The first is that although the literature shows that the presence of features suggestive of mandibular retropositioning are good predictors of OA success, in this study patients with balanced facial pattern benefited from treatment as well. This concept is of fundamental importance, as having a balanced pattern does not contraindicate treatment in populations with ESRD. The second important point is that individuals with skeletal changes may have an association of different maxillary and mandibular bone discrepancies, i.e., the same pattern II may be due to mandibular retrognathia and good maxillary positioning, or good mandibular positioning and maxillary protrusion, for example. Thus, one of the goals of this study was to identify these changes subjectively in order to eliminate unnecessary costs to the patient, exams that require radiation and optimize the relationship between the sleep physician and the dental surgeon in order to make a clinical diagnosis and clearly indicate the OA.

Among the limitations of this study, we can mention about the sample of type III facial patients. Our study was composed of only two type III patients and no short or long face. This may be explained by the fact that the prevalence of type I and II facial patterns is higher in the general population, in addition to the fact that therapy for type III facial pattern has a higher side effect rate and type III associated with maxillary hypoplasia still does not respond adequately to mandibular advancement, often being already excluded from the use of OA and directed to other types of treatment.

## Conclusion

Facial pattern types 1 and 2 did not influence the success of OA, and may benefit both facial patterns. The inter-rater agreement for the characterization of facial pattern types was considered “strong” and may be an auxiliary method in the evaluation of patients with OSA with indication for treatment with OA.

## ORCID IDs

Rafael de Andrade Balsalobre: 0009-0008-5332-0389

Maximiana Cristina de Souza Maliska: 0000-0003-1074-4313

Fernanda Louise Martinho Haddad: 0000-0002-5597-1176

## Funding

There is no funding source.

## Data availability statement

The authors declare that all data are available in repository.

## Declaration of competing interest

The authors declare no conflicts of interest.
